# Factors linked to the late diagnosis of breast cancer and the initiation of treatment

**DOI:** 10.11604/pamj.2024.47.207.42734

**Published:** 2024-04-24

**Authors:** Kalil Cissé, Ganiou Adjadé, Mohammed El Fadli, Ismail Essadi, Rhizlane Belbaraka

**Affiliations:** 1 Department of Medical Oncology, Mohammed VI University Hospital, University of Cady Ayyad, Marrakech, Morocco,; 2 Department of Onco Radiotherapy, Orleans University Hospital, Orleans, France,; 3 Department of Medical Oncology, Avicenna Military Hospital, University of Cady Ayyad, Marrakech, Morocco

**Keywords:** Breast cancer, late diagnosis, delays, prognosis, Marrakech, Morocco

## Abstract

Breast cancer is the first cancer in women in terms of incidence and mortality. In Morocco, it is a public health problem. Its prognosis is strongly linked to the stage at which it is diagnosed. It is a pathology for which diagnosis means are highly developed today, ranging from early detection to the demonstration of infra-clinical lesions, which has markedly improved the prognosis in developed countries. This work aims to identify the factors that lead patients to consult at an advanced stage in our daily practice. It is a retrospective study carried out from January 2018 to December 2018 including 525 patients with breast cancer followed in the medical oncology department of the Mohammed VI University Hospital in Marrakech. The average age was 54. The average time for consultation was 10.3 months. 63% of patients were from rural areas. Delayed diagnosis affected women above 35 years of age (80%). The most common method of detection was self-examination in 74% of cases. Inflammation (2.66%), ulceration (1.14%), signs of metastases (17.14%), and isolated breast nodes (79.4%) were other reasons for consultation. 82.2% of patients were locally advanced at the time of diagnosis. The time for treatment in our study was 3.7 weeks. In our practice, it is the conjunction of ignorance, poverty, socio-cultural habits, and difficult geographical access that are the essential factors in the late diagnosis of breast cancer.

## To the editors of the Pan African Medical Journal

Breast cancer is a public health problem around the world. 1.7 million cases of breast cancer appear each year and nearly 627,000 deaths [1]. Most deaths occur in low- and middle-income countries, where the diagnosis of breast cancer is very late, especially due to a lack of information on early detection and access insufficient in health services [2]. This cancer also affects men at a frequency of 3 to 4% in Africa [3]. It is the leading cause of female cancer death in Morocco. Its prognosis is closely linked to the stage of diagnosis. Diagnosis means are highly developed today, ranging from early detection to the demonstration of infra-clinical lesions, which has markedly improved the prognosis in developed countries. Despite significant advances in the fight against breast cancer, the time to diagnosis and access to treatment remains problematic in several countries. Measuring the delay appears to be a real element of the quality of care in addition to being a potential tracer of inequalities in access to care through this retrospective study occurring in the oncology department of Mohammed VI University Hospital. The main objective of our study is to evaluate the impact of the delay on the prognosis of patients followed for breast cancer, to reduce the inequalities in access to care that constitute possible delays.

Data were collected using a collection sheet entered into Microsoft Office Excel 2003 and then analyzed using SPSS version 21.0 software. From January 2018 to December 2018, we included 525 patients (60%). The average age was 54 with ranges of 28 to 79 years. The reasons for consultation were represented by ulceration, inflammation, metastatic signs, and isolated breast nodules. 82.2% of patients were locally advanced at the time of diagnosis and 17.1% were at a metastatic stage. The most frequent mode of detection was self-examination in 74% of cases. Regarding the factors identified to justify the delay, the questioning revealed: the lack of financial means in 12.8% of cases, the distance from hospitals in 61.9% of cases, socio-cultural habits with traditional treatments in 13.1% of cases, and insufficient social coverage in 12.2% of cases.

However, taken individually, no significant agreement was found between these factors and the long diagnosis delay. 36% of patients were from urban areas compared to 63% from rural areas and the average delay was 10.3 months. Women above 35 years of age were most affected (80%) by delayed diagnosis, where 86.8% experienced a delay >10 months, and 73.1% between 6 and 10 months. More than half of the patients were non-working (64.8%) and were most affected by the delayed diagnosis. These patients represent 68.9% of delays>10 months and 73.1% of delays between 6 and 10 months. The average delay in breast cancer diagnosis was 10.3 months with a time for treatment in our study of 3.7 weeks. We noticed that the clinical presentation was associated with delay, which is confirmed by the literature [4,5]. In general, patients are unaware of the first symptom and wait for the appearance of other symptoms or downright aggravation of the health condition before consulting [4,5]. Also, we found that Patients with aggressive types of breast cancer, especially those revealed by progressive symptoms in a short time such as skin inflammatory signs or necrotic ulceration, are most associated with a low rate of delayed diagnosis as found in other studies [6].

In our study, older women waited longer than younger women before consulting a physician, (86.8% of delays >10 months and 73.1% of delays between 6 and 10 months were >35 years old). Which was reported in some studies [4]. Concerning the impact of professional status, our study found that non-working patients (64.8%) were most affected by delayed diagnosis (68.9% of delays>10 months and 73.1% of delays between 6 and 10 months). The literature disagrees on this point. A study found no significant association between employment and delay in cancer diagnosis, in contrast to studies suggesting that employment was a barrier to delayed breast cancer diagnosis [7]. Through this study, we have demonstrated that the delay attributable to the patient (ignorance, indigence, socio-cultural habit) and to the health system played a role in the diagnosis delay. These delays call for the identification and implementation of interventions to reduce the delays and allow for an early diagnosis to improve the prognosis and patients´ quality of life (Table 1).

**Table 1:** distribution of diagnostic delay according to incriminated factors, patient age, and the professional status of patients

Diagnosis delay	<6 months	6-10 months	> 10 months	Numbers	Percentage (%)
Lack of financial means	30	20	17	67	12.8
Distance	110	109	106	325	61.9
Traditional habit	20	23	26	69	13.1
Insufficient support	27	19	18	64	12.2
Numbers (%)	187(35.6)	171(32.6)	167(31.8)	525	
Age<35	37	46	22	105	20
Age >35	150	125	145	420	80
Numbers (%)	187(35.6)	171(32.6)	167(31.8)	525	
Working patients	87	46	52	185	35.2
Non-working patients	100	125	115	340	64.8
Numbers (%)	187(35.6)	17(32.6)	167(31.8)	525	

## Conclusion

It is therefore essential to control this waiting time during care to ensure the success of the treatment. Efforts must be directed towards good information for the population at risk, better accessibility to early screening, the fight against poverty, and continuous medical training to enable early diagnosis and treatment

## References

[1] IARC Globocan Latest global cancer data: Cancer burden rises to 18.1 million new cases and 9.6 million cancer deaths in 2018. IARC Globocan.

[2] Price AJ, Ndom P, Atenguena E, Mambou Nouemssi JP, Ryder RW (2012). Cancer care challenges in developing countries. Un cancer.

[3] Ndom P, Um G, Bell EM, Eloundou A, Hossain NM, Huo D (2012). A meta-analysis of male breast cancer in Africa. Breast.

[4] Ermiah E, Abdalla F, Buhmeida A, Larbesh E, Pyrhönen S, Collan Y (2012). Diagnosis delay in Libyan female breast cancer. BMC Res Notes.

[5] Norsa'adah B, Rampal KG, Rahmah MA, Naing NN, Biswal BM (2011). Diagnosis delay of breast cancer and its associated factors in Malaysian women. BMC Cancer.

[6] Grunfeld EA, Hunter MS, Ramirez AJ, Richards MA (2003). Perceptions of breast cancer across the lifespan. J Psychosom Res.

[7] Limam M, Ajmi T, Zedini C, Khelifi A, Mellouli M, El Ghardallou M (2016). Etude de délais de traitement cancer du sein à Sousse (Tunisie). Santé Publique.

